# Supplementary figures and images for: Potentially inappropriate prescribing in older adults with advanced chronic kidney disease
Source: PLoS One. 2020 Aug 20;15(8):e0237868. doi: 10.1371/journal.pone.0237868 (PMC7444541; doi:10.1371/journal.pone.0237868)

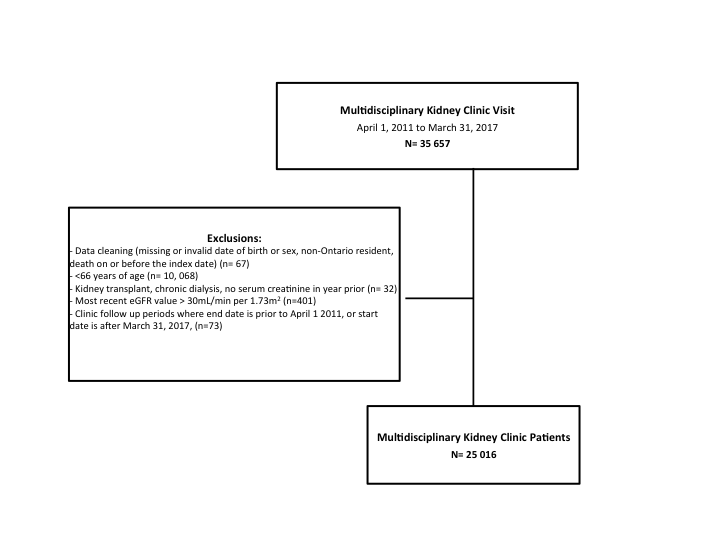

Supplement: S1 Fig — (TIFF) [file pone.0237868.s001.tiff]

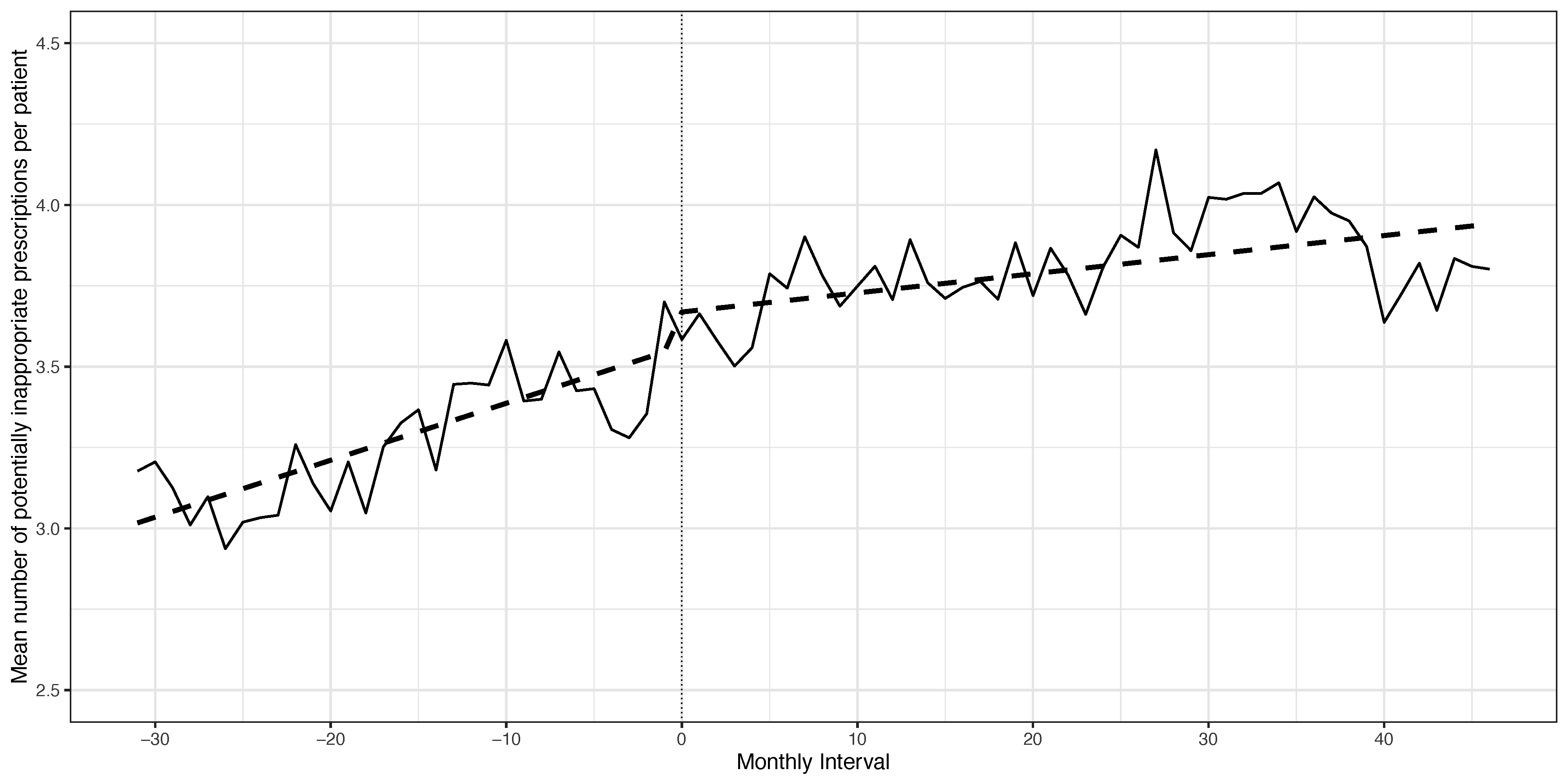

Supplement: S2 Fig — (TIFF) [file pone.0237868.s002.tiff]
