# Supplementary material for: Potentially inappropriate prescribing in older adults with advanced chronic kidney disease
Source: PLoS One. 2020 Aug 20;15(8):e0237868. doi: 10.1371/journal.pone.0237868 (PMC7444541; doi:10.1371/journal.pone.0237868)
Supplement: S1 Table — (DOCX) [file pone.0237868.s003.docx]

**S1 Table: Cumulative incidence of medications recommended to be avoided in patients with an eGFR <15 mL/min/1.73 m^2a^**

| **Medication** | **Cumulative incidence** |
| --- | --- |
| Apixaban | 33/5689 (0.6%) |
| Dabigatran | 6/5689 (0.1%) |
| Rivaroxaban | 0/5689 (0.0%) |
| Fibrates | 71/5689 (1.3%) |
| Glyburide | 85/5689 (1.5%) |
| SGLT-2 inhibitor^b^ | ≤5/5689 (<0.1%) |
| Nitrofurantoin | 407/5689 (7.2%) |
| Baclofen | 71/5689 (1.3%) |
| Metformin | 125/5689 (2.2%) |
| Duloxetine | 15/5689 (0.3%) |

^a^Examined in sub-group of patients with a baseline eGFR <15 mL/min/1.73 m^2^

^b^SGLT-2 inihibitor: Sodium glucose transporter-2 inhibitor (dapagliflozin, canagliflozin, empagliflozin)

Note: In accordance with ICES privacy policies, cell sizes less than or equal to five cannot be reported.
