# Supplementary material for: Potentially inappropriate prescribing in older adults with advanced chronic kidney disease
Source: PLoS One. 2020 Aug 20;15(8):e0237868. doi: 10.1371/journal.pone.0237868 (PMC7444541; doi:10.1371/journal.pone.0237868)
Supplement: S2 Table — (DOCX) [file pone.0237868.s004.docx]

**S2 Table: Cumulative incidence of medications dispensed above the recommended dose for an eGFR <30 mL/min/1.73 m^2^**

| **Medication** | **Cumulative incidence** |
| --- | --- |
| Acyclovir, where dose is >800 mg 3x/day | 17/25,016 (0.1%) |
| Apixaban, where age is >80 and dose is >2.5mg 2x/day | 33/9404 (0.4%) |
| Rivaroxaban, where dose is >15 mg daily | 0/25,016 (0.0%) |
| Ciprofloxacin, where dose is >500 mg every 12 hours | 112/25,016 (0.5%) |
| Levofloxacin, where dose is ≥500 mg daily | 1,188/25,016 (4.8%) |
| Valacyclovir, where dose is >1g daily | 0/25,016 (0.0%) |
