# Supplementary material for: Potentially inappropriate prescribing in older adults with advanced chronic kidney disease
Source: PLoS One. 2020 Aug 20;15(8):e0237868. doi: 10.1371/journal.pone.0237868 (PMC7444541; doi:10.1371/journal.pone.0237868)
Supplement: S3 Table — (DOCX) [file pone.0237868.s005.docx]

**S3 Table: Baseline characteristics pre-and post-pharmacist introduction^a^**

| **Characteristic** | **Pre-intervention Period**  **N=267** | **Post-Intervention Period**  **N=822** | **Standardized Difference^b^** |
| --- | --- | --- | --- |
| **Demographics** | | | |
| Age | 77 (6.7) | 77 (7.0) | 0.04 |
| Sex (male) | 167 (62.5) | 488 (59.4) | 0.06 |
| Income quintile |  |  |  |
| 1 (lowest) | 57 (21.3) | 204 (24.8) | 0.08 |
| 2 | 63 (23.6) | 174 (24.8) | 0.06 |
| 3 | 47 (17.6) | 148 (18.0) | 0.01 |
| 4 | 48 (18.0) | 149 (18.1) | 0.00 |
| 5 (highest) | 45 (16.9) | 129 (15.7) | 0.03 |
| Missing | 7 (2.6) | 18 (2.2) | 0.03 |
| Rural Location^c^ | 39 (14.6) | 93 (11.3) | 0.10 |
| **Comorbidities**^d^ | | | |
| Atrial fibrillation | 39 (14.6) | 136 (16.5) | 0.05 |
| Chronic obstructive pulmonary disease | 22 (8.2) | 70 (80.5) | 0.01 |
| Congestive heart Failure | 51 (19.1) | 178 (21.7) | 0.06 |
| Diabetes | 166 (62.2) | 550 (66.9) | 0.10 |
| Hypertension | 260 (97.4) | 775 (94.3) | 0.16 |
| Myocardial infarction | 23 (8.6) | 80 (9.7) | 0.04 |
| Peripheral vascular disease | 11 (4.1) | 34 (4.1) | 0.00 |
| **Kidney Function**^e^ | | | |
| Serum creatinine (μmol/L) | 299.7 (90.6) | 315.9 (127.5) | 0.15 |
| eGFR (mL/min/1.73 m^2^) | 16.4 (5.2) | 16.2 (6.0) | 0.04 |
| eGFR < 15 (mL/min/1.73 m^2^) | 125 (46.8) | 363 (44.2) | 0.05 |
| Urine albumin to creatinine ratio (mg/mmol) | 61.4 (88.7)^f^ | 109.7 (145.9)^g^ | 0.40 |

^a^Patient characteristics at the first vs. last interval were compared. Continuous characteristics are reported as mean (standard deviation, SD) and categorical characteristics are reported as proportions (%).^b^Standardized differences are less sensitive to sample size than traditional hypothesis tests. They provide a measure of difference between groups with respect to a pooled standard deviation. A standardized difference ≥0.1 is considered a meaningful difference between groups. ^c^Rural defined as residing in a location with a population of ≤10 000 individuals. ^d^Comorbidities in the past 5 year were considered.  ^e^Laboratory measurements in the past 1 year were considered. The most recent value was used. eGFR was determined using the CKD-EPI equation. ^f^Missing values, n=152 (57%). ^g^Missing values, n=153 (19%).
