# Supplementary material for: Potentially inappropriate prescribing in older adults with advanced chronic kidney disease
Source: PLoS One. 2020 Aug 20;15(8):e0237868. doi: 10.1371/journal.pone.0237868 (PMC7444541; doi:10.1371/journal.pone.0237868)
Supplement: S4 Table — (DOCX) [file pone.0237868.s006.docx]

**S4 Table: Change point regression analysis examining proportion of patients with potentially inappropriate prescribing pre-and post-pharmacist introduction**

| **Time interval** | **Estimate** | **Standard Error** | **p-value** |
| --- | --- | --- | --- |
| Pre-pharmacist introduction | 0.112 | 0.024 | < 0.001^a^ |
| At pharmacist introduction | -0.809 | 0.540 | 0.14^b^ |
| Post-pharmacist introduction | -0.116 | 0.028 | < 0.001^c^ |

^a^Change in potentially inappropriate prescribing per monthly interval pre-pharmacist introduction

^b^Change in the intercept pre- vs. post-pharmacist introduction

^c^Change in the slope post-pharmacist compared to pre-pharmacist introduction
