# Supplementary material for: Potentially inappropriate prescribing in older adults with advanced chronic kidney disease
Source: PLoS One. 2020 Aug 20;15(8):e0237868. doi: 10.1371/journal.pone.0237868 (PMC7444541; doi:10.1371/journal.pone.0237868)
Supplement: S7 Table — (DOCX) [file pone.0237868.s009.docx]

**S7 Table: Change point regression analysis examining the mean number of potentially inappropriate prescriptions per patient pre-and post-pharmacist introduction**

| **Time** | **Estimate** | **Standard Error** | **p-value** |
| --- | --- | --- | --- |
| Pre-pharmacist introduction | 0.018 | 0.004 | < 0.0001^a^ |
| At pharmacist introduction | 0.118 | 0.085 | 0.17^b^ |
| Post-pharmacist introduction | -0.012 | 0.005 | 0.012^c^ |

^a^Change in potentially inappropriate prescribing per monthly interval pre-pharmacist introduction

^b^Change in the intercept pre- vs. post-pharmacist introduction

^c^Change in the slope post-pharmacist compared to pre-pharmacist introduction
