# Supplementary material for: Potentially inappropriate prescribing in older adults with advanced chronic kidney disease
Source: PLoS One. 2020 Aug 20;15(8):e0237868. doi: 10.1371/journal.pone.0237868 (PMC7444541; doi:10.1371/journal.pone.0237868)
Supplement: S1 File — (DOCX) [file pone.0237868.s010.docx]

Appendix A. Checklist of recommendations for reporting of observational studies using the REporting of studies Conducted using Observational Routinely-collected health Data (RECORD) Statement

|  | **Item No** | **STROBE items** | **RECORD items** | **Reported** |
| --- | --- | --- | --- | --- |
| **Title and abstract** | 1 | (a) Indicate the study's design with a commonly used term in the title or the abstract.  (b) Provide in the abstract an informative and balanced summary of what was done and what was found. | (1.1) The type of data used should be specified in the title or abstract. When possible, the name of the databases used should be included.  (1.2) If applicable, the geographic region and time frame within which the study took place should be reported in the title or abstract.  (1.3) If linkage between databases was conducted for the study, this should be clearly stated in the title or abstract. | Abstract  Abstract  Abstract |
| **Introduction** |  |  |  |  |
| Background/ rationale | 2 | Explain the scientific background and rationale for the investigation being reported. |  | Introduction |
| Objectives | 3 | State specific objectives, including any prespecified hypotheses. |  | Introduction |
| **Methods** |  |  |  |  |
| Study design | 4 | Present key elements of study design early in the paper. |  | Methods: Design and Setting |
| Setting | 5 | Describe the setting, locations, and relevant dates, including periods of recruitment, exposure, follow-up, and data collection. |  | Methods: Design and Setting, Data Sources, Study Cohort |
| Participants | 6 | (a) Give the eligibility criteria, and the sources and methods of selection of participants. Describe methods of follow-up.  (b) For matched studies, give matching criteria and number of exposed and unexposed. | (6.1) The methods of study population selection (such as codes or algorithms used to identify subjects) should be listed in detail. If this is not possible, an explanation should be provided.  (6.2) Any validation studies of the codes or algorithms used to select the population should be referenced. If validation was conducted for this study and not published elsewhere, detailed methods and results should be provided.  (6.3) If the study involved linkage of databases, consider use of a flow diagram or other graphical display to demonstrate the data linkage process, including the number of individuals with linked data at each stage. | Methods: Study Cohort  S1 Fig, Appendix C  n/a  S1 Fig |
| Variables | 7 | Clearly define all outcomes, exposures, predictors, potential confounders, and effect modifiers. Give diagnostic criteria, if applicable. | (7.1) A complete list of codes and algorithms used to classify exposures, outcomes, confounders, and effect modifiers should be provided. If these cannot be reported, an explanation should be provided. | Appendix C, D and E |
| Data sources/   measurement | 8 | For each variable of interest, give sources of data and details of methods of assessment (measurement). Describe comparability of assessment methods if there is more than one group. |  | Methods: Data Sources, Supplemental Appendix B, C  n/a |
| Bias | 9 | Describe any efforts to address potential sources of bias. |  | Methods |
| Study size | 10 | Explain how the study size was arrived at. |  | Methods: Study Cohort, S1 Fig |
| Quantitative variables | 11 | Explain how quantitative variables were handled in the analyses. If applicable, describe which groupings were chosen and why. |  | Methods: Statistical Analyses |
| Statistical methods | 12 | (a) Describe all statistical methods, including those used to control for confounding.  (b) Describe any methods used to examine subgroups and interactions.  (c) Explain how missing data were addressed.  (d) If applicable, explain how loss to follow-up was addressed.  (e) Describe any sensitivity analyses. |  | Methods: Statistical Analyses |
| Data access and cleaning methods |  | N/A | (12.1) Authors should describe the extent to which the investigators had access to the database population used to create the study population.  (12.2) Authors should provide information on the data cleaning methods used in the study. | Methods: Design and Setting  Data Access/Access to Data Analysis Protocol  Methods: Study Cohort |
| Linkage |  | N/A | (12.3) State whether the study included person-level, institutional-level, or other data linkage across two or more databases. The methods of linkage and methods of linkage quality evaluation should be provided. | Methods: Design and Setting |
| **Results** |  |  |  |  |
| Participants | 13 | (a) Report numbers of individuals at each stage of study--e.g. numbers potentially eligible, examined for eligibility, confirmed eligible, included in the study, completing follow-up, and analyzed.  (b) Give reasons for non-participation at each stage.  (c) Consider use of a flow diagram. | (13.1) Describe in detail the selection of the persons included in the study (i.e., study population selection), including filtering based on data quality, data availability, and linkage. The selection of included persons can be described in the text and/or by means of the study flow diagram. | S1 Fig |
| Descriptive data | 14 | (a) Give characteristics of study participants (e.g. demographic, clinical, social) and information on exposures and potential confounders.  (b) Indicate number of participants with missing data for each variable of interest.  (c) Summarize follow-up time (e.g. average and total amount). |  | Results, Table 1, Supplementary Table 3 |
| Outcome data | 15 | Report numbers of outcome events or summary measures over time. |  | Results, Table 2 |
| Main results | 16 | (a) Give unadjusted estimates and, if applicable, confounder-adjusted estimates and their precision (e.g. 95% confidence interval). Make clear which confounders were adjusted for and why they were included.  (b) Report category boundaries when continuous variables were categorized.  (c) If relevant, consider translating estimates of relative risk into absolute risk for a meaningful time period. |  | Results, Table 2, Figures 1, 2, 3  Supplementary Tables 1, 2, 4-6 |
| Other analyses | 17 | Report other analyses done (e.g. analyses of subgroups and interactions, and sensitivity analyses). |  | Results, Supplementary Table 7, S2 Fig |
| Key results | 18 | Summarize key results with reference to study objectives. |  | Discussion |
| Limitations | 19 | Discuss limitations of the study, taking into account sources of potential bias or imprecision. Discuss both direction and magnitude of any potential bias. | (19.1) Discuss the implications of using data that were not created or collected to answer the specific research question(s). Include discussion of misclassification bias, unmeasured confounding, missing data, and changing eligibility over time, as they pertain to the study being reported. | Discussion |
| Interpretation | 20 | Give a cautious overall interpretation of results considering objectives, limitations, multiplicity of analyses, results from similar studies, and other relevant evidence. |  | Discussion/Conclusions |
| Generalizability | 21 | Discuss the generalizability (external validity) of the study results. |  | Discussion |
| **Other information** | |  |  |  |
| Funding | 22 | Give the source of funding and the role of the funders for the present study and, if applicable, for the original study on which the present article is based. |  | Acknowledgment: Sources of Funding |
| Accessibility of protocol, raw data, and programming code |  | N/A | (22.1) Authors should provide information on how to access any supplemental information such as the study protocol, raw data, or programming code. | Data Access/Access to Data Analysis Protocol |

*Reference: Benchimol EI, Smeeth L, Guttmann A, Harron K, Moher D, Petersen I, Sørensen HT, von Elm E, Langan SM, the RECORD Working Committee. The REporting of studies Conducted using Observational Routinely-collected health Data (RECORD) Statement. *PLoS Medicine* 2015 Oct 6;12(10):e1001885.

*Checklist is protected under Creative Commons Attribution ([CC BY](http://creativecommons.org/licenses/by/4.0/)) license.

| Appendix B: Description of ICES Data Sources | |
| --- | --- |
| **Database** | **Description** |
| Canadian Institute for Health Information’s Discharge Abstract Database/ Same Day Surgery | Database contains diagnostic and procedural information for all hospitalizations. |
| Ontario Hypertension Dataset | ICES derived database that contains all individuals with hypertension |
| National Ambulatory Care Reporting System | Database contains information on hospital and community based ambulatory care visits. |
| Ontario Drug Benefits | Database contains highly accurate records of all dispensed outpatient prescriptions covered through the Ontario Drug Benefits program. |
| Ontario Diabetes Dataset | ICES derived database that contains all individuals with any type of non-gestational diabetes |
| Ontario Health Insurance Plan | Database includes diagnostic information, and health claims for inpatient and outpatient services. |
| Ontario Renal Reporting System | Database contains chronic kidney disease and renal dialysis related information |
| Ontario Laboratories Information System | Database contains laboratory test orders and results from hospitals, community labs, and public health labs. |

**Appendix C: Codes used in the study to identify cohort inclusion, exclusion, and baseline comorbid conditions**

| **Cohort Inclusion** | | |
| --- | --- | --- |
| Eligible MCKC Patients | | |
| ORRS |  | 1. PTTREATEVENT_R6_2016 [StatusCD] = "O"  2. PTTREATEVENT_R6_2016 [MODALITYID]= "65" and [TREATMENTCHANGECD]= "NP" or "VR" and  PTPREDLYSSRVCDETAIL[CLINICVISITID]= "1" |
| **Cohort Exclusion** | | |
| Death | | |
| RPDB |  | DTHDATE |
| Age < 66 | | |
| RPDB |  | % getdemo ICES macro |
| Kidney Transplant | | |
| CIHI-DAD | CCI | “IPC85” |
| OHIP | Fee code | "S435", "S434" |
| Chronic Dialysis | | |
| ORRS |  | PATIENT_TREATMENT_EVENT dataset  [STATUSCD]: "O"  [TREATMENTCHANGECD]:"N"  [TREATMENTDATE]  PATIENT_TREATMENT_EVENT dataset    [MODALITYID]: “1”, “12”, “13”, “14”, “15”, “16”, “18”, “21”, “22”, “26”, “33”, “44”, “86”, “85”, “8”, “10”, “17”, “19”, “20”, “24”, ”25”, “28”, ”29”, ”57”, ”58”, “76”, “80”, “81”, “87”, “88”, “89” ,”90”, “92”, “93”, “2”, “23”, “55”, “56”, “59”, “60”, “77”, “78”, “79” |
| CIHI-DAD | ICD10 | "Z49", "Z992" |
|  | CCI | "1PZ21" |
| OHIP | Fee code | "R849", "G323", "G325", "G326", "G860", "G862", "G865" "G863", "G866", "G330", "G331", "G333", "G861", "G082", "G083", "G085", "G090", "G091", "G092", "G093", "G094", "G095", "G096", "G294", "G295", "G864", "H540", "H740" |
| **Comorbid Conditions** | | |
| Atrial Fibrillation | | |
| CIHI-DAD | ICD10 | "I48" |
| OHIP | Diagnostic code | "427" |
| Chronic Obstructive Pulmonary Disease | | |
| CIHI-DAD | ICD10 | "J41", "J43", "J44" |
| Congestive Heart Failure | | |
| CIHI-DAD | ICD10 | "I099", "I420", "I425", "I426", "I427", "I428", "I429", "I43", "I500", "I501", "I509", "I255", "J81" |
|  | CCI | "1HP53", "1HP55", "1HZ53GRFR", "1HZ53LAFR", "1HZ53SYFR" |
| OHIP | Diagnostic code | "428" |
|  | Fee code | "R701", "R702", "Z429" |
| Diabetes | | |
| ODD |  | DIAGDATE, PREV2017 |
| Hypertension | | |
| HYPER |  | DIAGDATE, PREV2017 |
| Myocardial Infarction | | |
| CIHI-DAD | ICD10 | "I21", "I22" |
| Peripheral Vascular Disease | | |
| CIHI-DAD | ICD10 | "I700", "I702", "I708", "I709", "I731", "I738", "I739", "K551" |
|  | CCI | "1KA76", "1KA50", "1KE76", "1KG50", "1KG57", "1KG76MI", "1KG87", "1IA87LA", "1IB87LA", "1IC87LA", "1ID87", "1KA87LA", "1KE57" |
| OHIP | Fee code | "R787", "R780", "R797", "R804", "R809", "R875", "R815", "R936", "R783", "R784","R785", "E626", "R814", "R786", "R937", "R860", "R861", "R855", "R856", "R933", "R934", "R791", "E672", "R794", "R813", "R867", "E649" |
| Benign Prostatic Hyperplasia | | |
| CIHI-DAD | ICD10 | “N40”, “D291”, “R33”, “R350”, “R358”, “R3910”, “R3911”, “R3912”, “R3913”, “R3914”, “R3918” |
| OHIP | Fee code | “S655”, “S654” |

Database Abbreviations: CIHI-DAD-Canadian Institutes for Health Information’s Discharge Abstract Database,

CCI-Canadian Classification of Interventions, HYPER- Ontario Hypertension Database, ODD- Ontario Diabetes Dataset,

OHIP- Ontario Health Insurance Plan, ORRS- Ontario Renal Reporting System, RPDB- Registered Persons Database,

## Appendix D: List of Potentially Inappropriate Prescriptions

| **Drug Name or Class** |
| --- |
| *i. Presence of the following:* |
| Colchicine, Lithium, Acyclovir, Digoxin, Apixaban, Dabigatran, Rivaroxaban,  Spironolactone, Methotrexate, Fibrates, Morphine, Glyburide, Canagliflozin, Dapagliflozin, Empagliflozin, Ciprofloxacin, Levofloxacin, Nitrofurantoin, Baclofen, Valacyclovir, Metformin, Pregabalin, Gabapentin, Duloxetine, Peripheral alpha blockers, Alpha agonists, Tricyclic antidepressants, Benzodiazepines, Proton pump inhibitor *(Note: >8 weeks duration),* Skeletal muscle relaxants, Codeine, Metoclopramide, First generation antihistamines, Anti-arrhythmic drugs, Antipsychotics, Paroxetine |
| *ii. Absence of the following:* |
| Statins  *Note: Patients not prescribed statins are considered to be potentially inappropriately prescribed* |

## Appendix E: Potentially Inappropriate Prescription Categories

| **Category** | **Drug Name** |
| --- | --- |
| Medications of concern in CKD | Colchicine, Lithium, Acyclovir, Digoxin, Apixaban, Dabigatran, Rivaroxaban, Spironolactone, Methotrexate, Fibrates, Morphine, Glyburide, Dapagliflozin, Canagliflozin, Empagliflozin, Ciprofloxacin, Levofloxacin, Nitrofurantoin, Baclofen, Valacyclovir, Metformin, Pregabalin, Gabapentin, Duloxetine |
| Medications of concern in older patients | Digoxin (Note: where dose is > 0.125mg/day), Glyburide, Peripheral alpha blockers, Alpha agonists, Tricyclic antidepressants, Benzodiazepines, Proton pump inhibitors (Note: >8 weeks duration), Skeletal muscle relaxants, Codeine, Metoclopramide, First generation antihistamines, Anti-arrhythmic drugs, Spironolactone (Note: where dose > 25mg/day), Antipsychotics, Paroxetine |
| Medications dispensed above the recommended dose for eGFR <30 mL/min/1.73 m^2^ | Acyclovir, where dose is > 800 mg 3x/day  Apixaban, where age is > 80 and dose is > 2.5mg 2x/day  Rivaroxaban, where dose is > 15 mg once daily  Ciprofloxacin, where dose is > 500 mg every 12 hours  Levofloxacin, where dose is ≥500 mg daily  Valacyclovir, where dose is > 1g daily  Note: *Daily dose = quantity dispensed x strength / days supply* |
| Medications recommended to be avoided at eGFR <15 mL/min/1.73 m^2^ | Apixaban, Dabigatran, Rivaroxaban, Fibrates, Glyburide, Dapagliflozin, Canagliflozin, Empagliflozin, Nitrofurantoin, Baclofen, Metformin, Duloxetine |
